# Globular cluster formation and evolution in the context of cosmological galaxy assembly: open questions

**DOI:** 10.1098/rspa.2017.0616

**Published:** 2018-02-14

**Authors:** Duncan A. Forbes, Nate Bastian, Mark Gieles, Robert A. Crain, J. M. Diederik Kruijssen, Søren S. Larsen, Sylvia Ploeckinger, Oscar Agertz, Michele Trenti, Annette M. N. Ferguson, Joel Pfeffer, Oleg Y. Gnedin

**Affiliations:** 1 Centre for Astrophysics and Supercomputing, Swinburne University, Hawthorn Victoria 3122, Australia; 2 Astrophysics Research Institute, Liverpool John Moores University, 146 Brownlow Hill, Liverpool L3 5RF, UK; 3 Department of Physics, University of Surrey, Guildford GU2 7XH, UK; 4 Astronomisches Rechen-Institut, Zentrum für Astronomie der Universität Heidelberg, Monchhofstraße 12-14, 69120 Heidelberg, Germany; 5 Department of Astrophysics/IMAPP, Radboud University, PO Box 9010, 6500 GL Nijmegen, The Netherlands; 6 Leiden Observatory, Leiden University, PO Box 9513, 2300 RA Leiden, The Netherlands; 7 Lund Observatory, Department of Astronomy and Theoretical Physics, Lund University, PO Box 43, 22100 Lund, Sweden; 8 School of Physics, The University of Melbourne, Victoria 3010, Australia; 9 Institute for Astronomy, University of Edinburgh, Blackford Hill, Edinburgh EH9 3HJ, UK; 10 Department of Astronomy, University of Michigan, Ann Arbor, MI 48109, USA

**Keywords:** globular clusters: galaxy formation, cosmology, simulations

## Abstract

We discuss some of the key open questions regarding the formation and evolution of globular clusters (GCs) during galaxy formation and assembly within a cosmological framework. The current state of the art for both observations and simulations is described, and we briefly mention directions for future research. The oldest GCs have ages greater than or equal to 12.5 Gyr and formed around the time of reionization. Resolved colour-magnitude diagrams of Milky Way GCs and direct imaging of lensed proto-GCs at *z*∼6 with the James Webb Space Telescope (JWST) promise further insight. GCs are known to host multiple populations of stars with variations in their chemical abundances. Recently, such multiple populations have been detected in ∼2 Gyr old compact, massive star clusters. This suggests a common, single pathway for the formation of GCs at high and low redshift. The shape of the initial mass function for GCs remains unknown; however, for massive galaxies a power-law mass function is favoured. Significant progress has been made recently modelling GC formation in the context of galaxy formation, with success in reproducing many of the observed GC-galaxy scaling relations.

## 1. Preamble

A small group of researchers were invited by the Royal Society to attend a workshop at Chicheley Hall, Buckinghamshire. Over the course of 2 days (5, 6 April 2017), they presented new results and discussed globular cluster (GC) formation and evolution in the context of galaxy assembly. The article that follows represents some of the discussion from that meeting with a focus on the open questions that were raised and the prospects for answering those questions in the near future. We hope that this article will be of value to other researchers in this field, as well as those in related areas of research.

## 2. When did globular clusters form?

Knowing the age of GCs, and hence their redshift of formation for a given cosmology, is a key parameter for understanding their relationship to galaxy formation and for testing model predictions. For example, did GCs form more than 12.8 Gyr ago, i.e. before the end of reionization (*z*≈6) and perhaps play a role in reionizing the universe, or did they mostly form at later times closer to the peak in the cosmic star formation rate (*z*≈2)? Coupled with GC metallicities, knowing the age of GCs provides a stringent constraint on models of GC formation (e.g. [1–4]).

Almost 70 Milky Way GCs have age measurements based on deep colour-magnitude diagrams (CMDs) observed with the ACS onboard the Hubble Space Telescope. The resulting age-metallicity relation reveals a dominant population of very old GCs covering a wide range of metallicities and a ‘young’ branch for which age and metallicity are anti-correlated [5–8]. These studies generally find the very old metal-poor (MP) subpopulation to be somewhat older than the metal-rich (MR) subpopulation (i.e. approx. 12.5 versus approx. 11.5 Gyr, respectively), but coeval ages cannot yet be ruled out within the uncertainties. The ‘young branch’ GCs can be largely associated with the disrupted Sgr dwarf and other possible accreted GCs. We note that only a handful of Milky Way GCs have their ages determined from the white dwarf cooling sequence (e.g. [9,10]) which gives ages consistent with those measured from main sequence fitting. The method has the advantage of being less sensitive to metallicity, but it requires very deep CMDs.

What are the prospects for getting more age measurements of Milky Way GCs? It would be very useful to increase the current sample of HST-observed Milky Way GCs, particularly MR bulge GCs which are deficient in the current sample, largely due to foreground reddening issues. A new technique, which will help to address this issue, is to derive the absolute ages of GCs based on deep near-infrared CMDs that probe the main sequence ‘kink’. In the near-infrared, the CMDs of old stellar populations display a ‘kink’ at approximately 0.5 *M*_⊙_ (corresponding to J–K ∼ 0.8) due to opacity effects of H_2_. The magnitude difference between the main sequence turn-off and this kink is sensitive to age, and largely insensitive to uncertainties in the extinction and distance modulus as well as uncertainties in the stellar models [11]. The location of the kink is also dependent on the metallicity, but there are hopes that with precise James Webb Space Telescope (JWST) photometry, absolute ages will be possible to slightly less than 1 Gyr accuracy (e.g. [12,13]). It will also be interesting to determine relative ages for GCs with similar metallicities, which will provide a strong constraint on the timescale over which (at fixed metallicity) the GC system of the Milky Way was built up. An exciting possibility for future work with JWST is to measure the brown dwarf cooling sequence [14]. Although challenging, this method has the advantage of being insensitive to distance (a key element in the current error budget for GC age determinations).

We note that recently, six old LMC GCs were observed using ACS by Wagner-Kaiser *et al.* [15]. They find relative ages that are coeval with the old halo GCs of the Milky Way as measured by Marin-Franch *et al.* [5].

Age estimates for GCs beyond the Local Group come from integrated light. Spectra with moderate signal-to-noise ratio were obtained in the early 2000s with LRIS on the Keck 10 m (e.g. [16], GMOS on Gemini 8m (e.g. [17]) and FORS2 on the VLT 8m (e.g. [18])). The situation was summarized in a meta-analysis in 2005 by Strader *et al.* [19]. They concluded that both MP and MR subpopulations were ‘no younger than their Galactic counterparts, with ages greater than or equal to 10 Gyr’. The absorption line index measurements from these spectra had values lower than predicted by 14-Gyr-old stellar population tracks (i.e. formally indicating ages greater than the age of the universe). This inconsistency prevented the derivation of *absolute* ages. On the other hand, measurement uncertainties prevented the detection of *relative* age differences of less than 1–2 Gyr. The observations have a bias to targeting brighter (more massive) GCs and those located in galaxy central regions which have a higher number density. Determining the relative ages of inner and outer (i.e. predominately *in situ* and accreted, see §3) GCs is desirable. In the last decade, there have been very few attempts to further measure the ages of GCs from spectra in galaxies beyond the Local Group.

An alternative method for estimating GC ages was presented by Forbes *et al.* [20], using measurements of GC mean metallicities and assuming that they followed a galaxy mass-metallicity relation [21] extrapolated in redshift (and beyond the current observations). With these caveats in mind, which may lead to systematic uncertainties of greater than or equal to 1 Gyr, the method indicates MR GC mean ages of 11.5 Gyr (*z*_form_=2.9) and MP ages of 12.5–12.8 Gyr (*z*_form_=4.8–5.9). If correct, this would place GC formation around the time of reionization, and continuing after the epoch of reionization. The age (epoch of formation) and predictions from various GC simulations pre-2015 are summarized in Forbes *et al.* [20]—none can be convincingly ruled out on the basis of current GC age measurements.

To directly measure mean relative ages for large numbers of extragalactic GCs requires the multiplexing advantages of a very wide-field spectrograph that operates at blue wavelengths on a large telescope. A spectrograph that meets these requirements is the Prime Focus Spectrograph (PFS) to be commissioned on the Subaru 8.2 m telescope in 2019. PFS has 2400 fibres that can be allocated over a 1.3° diameter field-of-view. It operates from 0.38 to 1.26 μm, thus covering the key absorption sensitive to age in old stellar populations. PFS will be able to obtain spectra for GCs in the inner and outer regions of nearby galaxies, and for several group/cluster galaxies in a single pointing. Careful background subtraction of the host galaxy and sky will be required. In addition, measuring mean relative ages from these spectra will require, at the very least, high S/N and the reduction of random errors from a large number of objects. Even for the nearest extragalactic GCs, this work will be very challenging on current 8–10 m class telescopes and is better suited to the new 20–40 m class telescopes. On the theoretical side, improvements are also needed in the modelling of integrated-light age diagnostics, e.g. the contribution of blue horizontal branch (HB) stars thermally pulsing red giant branch stars. The expansion of the stellar library to lower metallicity, as carried out recently by Villaume *et al.* [22], is also particularly important for application of the models to GCs.

As noted recently by Renzini [23], we are on the cusp of observing the formation of GCs directly at high redshift. Pioneering work by Johnson *et al.* [24] and Vanzella *et al.* [25,26] has used strong lensing amplification in distant (*z*∼ 2–6) galaxy clusters imaged by HST to measure a small number of compact objects with typical sizes of 16–140 pc and masses of a few × 10^6^ M_⊙_. Even if such sizes still greatly exceed those of average GCs observed in the local Universe, these objects have been interpreted as GCs in formation. For one source at *z*=3.1, Vanzella *et al.* [26] measured a dynamical mass consistent with its stellar mass, implying no evidence of a mini dark matter halo. For an object at *z*=6.1, they measured a size (approx. 20 *pc*) and mass (approx. 10^7^ M_⊙_) consistent with that expected for a proto-GC. Spectroscopy and imaging for large numbers of such compact objects at high redshift should be possible with JWST and help to determine if they are indeed proto-GCs.

## 3. Where did globular clusters form?

Current ideas for massive galaxy formation focus on the concept of two-phase galaxy formation. Cosmological simulations (e.g. [27–29]) indicate that large galaxies underwent an initial *in situ* phase of formation followed by mass growth via an *ex situ* (or accretion) phase. In these simulations, star formation can be tagged as occurring within the potential well/virial radius of the host primary galaxy (i.e. *in situ*) or within the satellite galaxy that is eventually accreted (i.e. *ex situ*). Thus in the simulations, *in situ* and *ex situ* formed material can be defined and tracked separately. These simulations predict that the most massive galaxies have accreted a large fraction of their final mass, while for the low mass galaxies their assembly is dominated by *in situ* star formation. This suggests that today's galaxies should contain contributions from both *in situ* and *ex situ* formed GCs depending on the galaxy's assembly history.

The distinction between *in situ* formed GCs and those that were accreted from another galaxy is much harder to discern observationally. Indeed, the concept itself is less well defined during the initial phase of galaxy formation at high redshift (which may involve clumpy dissipative collapse, and inflowing cold gas onto a chaotic, turbulent gas disc) and at later times when gas is involved (e.g. GCs may form in the primary galaxy from low-metallicity gas that was acquired from a satellite). Bearing these caveats in mind, an open question within the context of current galaxy formation models is: where did GCs form?

The stellar components of massive early-type galaxies and their red GCs have many properties in common (e.g. [30]). Thus, it is often assumed that the MR GCs are associated with the *in situ* phase of galaxy formation, and that MP GCs are all accreted. However, the situation for the MR and MP subpopulations may be more complex. Here, colour gradients in the subpopulations offer some clues. Radial colour gradients, in the sense that the innermost GCs are the most MR, have been detected in the MR and MP subpopulations separately in over a dozen GC systems (e.g. [31]). They are relatively weak and hence require high-quality data for a large sample of GCs to be detectable (which generally restricts galaxies beyond the Local Group to be massive ellipticals). These colour gradients correspond to metallicity gradients with average values of around −0.20 dex per dex. Within a given galaxy, the MR and MP subpopulations tend to have similar gradients [32]. There are hints that these gradients correlate with host galaxy mass [33]. At very large radii, the gradients appear to flatten out to a constant colour/metallicity (which, in turn, can be used to infer the typical mass of accreted galaxies; e.g. [34]).

If the inner GC metallicity gradients of both subpopulations are associated with the *in situ* phase of galaxy formation, then that process needs to produce GC subpopulations that have different spatial distributions and mean metallicities. An alternative possibility is that both MR and MP GCs were deposited into the inner regions by progressively more massive satellites (and their more MR GCs), thus building up metallicity gradients in both GC subpopulations. A combination of this accretion process and *in situ* formed GCs is also possible. For GCs in the outer halo regions, which show no radial colour gradients, a purely *ex situ*/accretion origin seems most likely. It will be interesting to see if future simulations that incorporate GCs can reproduce these behaviours in the blue and red GC colour gradients, and hence provide a deeper insight into the processes and locations of GC formation. It will also be useful to have more studies of GC systems and their host galaxies to large radii, looking for common features in their GC/field population colour gradients and surface density/brightness profiles.

Local Group galaxies provide a unique and complementary view of *in situ* and accreted GCs, although one should bear in mind that it is a single environment, with a limited mix of galaxy types and masses. For example, the Local Group does not contain any massive elliptical galaxies, typically found in denser environments, which are thought to have assembled a large fraction of their mass by accreting satellite galaxies (and their GCs). Within the Local Group, GCs either fully or partially resolve into stars—they can thus be readily identified on the basis of morphology alone, permitting much cleaner and more complete samples of GCs at large radii, and one can derive precise information about the constituent stars from their CMDs. Studies to date have provided a wealth of evidence that accretion has played a significant role in building the halo GC systems of both the Milky Way and M31.

Searle & Zinn [35] were the first to argue that some fraction of the Milky Way GC system has an external origin. They showed that GCs outside the solar circle exhibit no metallicity gradient, nor any correlation between HB morphology (taken as a proxy for age) and metallicity, and concluded that the outer halo GC system arose due to the merger and accretion of ‘protogalactic fragments’ in a slow chaotic fashion. Modern studies with much improved datasets have only strengthened this assertion, and indeed have placed it within the cosmological context of hierarchical structure formation.

Arguably, the best example of GC accretion within the Milky Way is provided by the Sgr dwarf galaxy. There is direct evidence that at least 5 GCs have been brought into the halo as part of this disrupting system, with several other promising candidates (e.g. [36–38]). While most of these are old, MP GCs, they also include some relatively young and MR objects such as Terzan 7 ([Fe/H]=−0.56) and Whiting 1 ([Fe/H]=−0.65). Thus, the Sgr dwarf is contributing *both* MP and MR GCs to the Milky Way halo, likely reflecting the age-metallicity relationship of the dwarf galaxy itself [37,6]. Less direct but still compelling evidence for GC accretion in the Milky Way comes from considering the distribution of metallicities, velocities, ages, HB morphologies and sizes of halo GCs (e.g. [5,6,39,40]). Unfortunately, the number of accreted GCs within the Milky Way today is highly uncertain, with estimates ranging from approximately 30–100 (e.g. [6,8,39,41]). Nonetheless, considering the total population of Milky Way GCs is approximately 160, these numbers already demonstrate that a substantial fraction (up to $\frac{2}{3}$) of the Milky Way's system is likely of an external origin.

In M31, the INT/WFC Survey, the SDSS and the Pan-Andromeda Archaeological Survey (PAndAS) have led to the discovery of more than 90 GCs in the outer halo of M31, lying at projected radii more than 25 kpc (e.g. [42–44]). It is fascinating to note that this represents roughly seven times as many GCs as in the comparable region of the Milky Way halo, and that many of the M31 halo GCs exhibit a striking correlation with faint stellar debris streams—‘smoking gun’ evidence that they have been accreted along with their now-disrupted host galaxies. Alignments between GCs and stellar streams are seen in both position and velocity space [45–47] and the analysis suggests that more than 60% of the outer halo M31 GC system has an accretion origin. Taken as a whole, the M31 halo GCs are remarkably uniform in colour and high-resolution spectroscopic follow-up of a small sample has indicated that most of these are MP, with [Fe/H]≲−1.5 [48,49]. Nonetheless, accreted GCs can often be distinguished on the basis of their rather red HBs, which when combined with their metallicities, can be explained by having only younger ages (e.g. [50]). Indeed, in this sense, the accreted members of the M31 GC system share similar characteristics to those in the halo of the Milky Way.

Recently, there have been concerted efforts to expand our knowledge of GCs in dwarf galaxies, as these represent the most likely donors of the accreted GCs in large galaxy halos. The Local Group has more than 80 dwarf galaxies (M_*V*_>−16), but only a handful of these are currently known to possess GCs. Dedicated searches have improved our census in several cases (e.g. [51,52]), while some dwarf galaxy GCs have been stumbled upon serendipitously (e.g. [53,54]). Recently, GCs have been found around very low mass dwarf satellite galaxies of the Milky Way and M31, i.e. Eridanus II (M_*V*_=−7.1; [55]) and And I (*M*_*V*_=−11.7; [56]). There is clearly much to be learned from the detailed study of GCs in the lowest mass galaxies (e.g. [57–59]), and from quantitative comparison of these objects with the suspected accreted GC populations in the Milky Way and M31 halos.

## 4. Are ancient globular clusters different from more recently formed globular clusters?

Given the clear mass and density differences between Galactic open and GCs, early work on the origin of star clusters invoked special conditions of the early Universe for the formation of GCs. The first detailed attempt to explain GC formation within a cosmological context was that of Peebles & Dicke [60], who noted that the typical masses of GCs are comparable with the Jeans mass shortly after recombination. Fall & Rees [61] later suggested that GCs might form as a result of thermal instabilities in collapsing protogalaxies. In both of these scenarios, GC formation was thus viewed as a special phenomenon of the early Universe, different from present-day star formation. The discovery of young ‘super star clusters’ in the local Universe with masses and density equal to or even far above GCs (e.g. [62]), shifted the focus to scenarios in which GCs form largely by normal star formation processes.

In recent years, the differences between stellar populations in GCs and lower mass open clusters, and in many cases, young/intermediate age clusters in the LMC/SMC, have been used as an argument that GCs may be unique after all. In particular, GCs host star-to-star abundance spreads in C, N, O, Al and He (a.k.a. multiple populations), whereas younger systems appear to be largely consist of a single population with homogeneous chemistry [63]. It should be noted that the phenomenon of multiple populations is present in both extremely MP (e.g. NGC 7078, NGC 7099; [64]) and MR GCs (e.g. 47 Tuc, NGC 6388). Recent work, however, has shown that multiple populations are, in fact, present in GCs down to an age of approximately 2 Gyr, *z*_form_≈0.17 [65–67]. While multiple populations have not been found below this age, it is doubtful that the GC formation process was significantly different before/after the *z*_form_≈0.17 boundary. The cause of this approximately 2 Gyr age threshold in the onset of multiple populations is still not clear. It is possible that the phenomenon only manifests itself below a certain stellar mass (which translates into a certain age if only the RGB is investigated). This could be due to the presence of only low-mass stars with abundance anomalies or possibly there is a so-far-unknown stellar evolutionary effect. For instance, the stellar mass boundary separating GCs with/without multiple populations straddles the boundary where stars have strong magnetic fields and also very different rotational properties.

Instead of adopting special conditions for GC formation, which would imply a difference between them and younger star clusters with very similar properties (mass, size, density, stellar populations), a simpler approach would be to look for a global model of massive cluster formation. Can such a model explain the plethora of GC population properties, their relation to their host galaxy and the propeties of young/intermediate massive clusters? There has been work in this direction, with some success (e.g. [68]). Additionally, there is ongoing work to include what is known about massive cluster formation, evolution and destruction based largely on young massive clusters in the local Universe, and in cosmological hydrodynamical simulations of galaxy assembly (e.g. [3,69,70]).

As mentioned earlier, recent studies using gravitational lensing have been able to resolve down to nearly GC scales of 20–40 pc [24,25] in galaxies at redshifts *z*=2−6, offering the possibility of seeing GC formation in action. While these studies are still preliminary, the properties of the observed star clusters are similar to those of Young Massive Clusters (YMCs) observed in nearby (i.e. low redshift) starburst galaxies in terms of their (unresolved) sizes, masses and relation to the star formation rate of their host galaxies. This suggests that, at least on the high-mass end, YMCs and proto-GCs may share similar formation mechanisms/properties. However, the detailed stellar population properties (e.g. presence of multiple populations or not) of these high-*z* star clusters will likely remain unknown for the foreseeable future.

## 5. What was the initial mass of a globular cluster system?

### (a) Stellar population considerations

The total integrated magnitude of the (halo) GCs in the Milky Way (here defined as those with [Fe/H]<−1) is about *M*_*V*_(*tot*)=−12.9 (using data from [71]). For a typical mass-to-light ratio of *Υ*_*V*_≈1.5 [72,73], this corresponds to a total mass of about 1.2×10^7^*M*_⊙_. The total mass of the stellar halo is estimated to be (4−7)×10^8^*M*_⊙_ for 1<*R*<40 kpc [74], so the Milky Way GC system currently accounts for about 2–3% of the stellar halo mass. However, because of dynamical evolution, GCs are expected to lose mass over time, and some clusters may have completely disrupted. It is thus likely that the initial mass of the GC population was significantly higher than the present-day observed value.

The mass functions of young star cluster populations are typically well described by power laws with a slope of ≈−2 at the low-mass end, and an exponential cut-off at the high-mass end, $dN/dM\propto M^{-2}\exp(-M/M_{c})$ (see the review of [75]). This is in contrast to the mass functions observed in old GC systems, which have a deficit of low-mass clusters with *dN*/*dM*≈ const. below some characteristic mass. When plotted in bins of log⁡(M), or absolute magnitude, a flat d*N*/d*M* gives rise to the familiar peaked form of the GC luminosity function with a peak at *M*_*V*_≈−7.5. The mass functions of old GCs can generally be quite well described by an *evolved* Schechter functions of the form [76]

5.1 $$\frac{dN}{dM}\propto {\left(M+\Delta M\right)}^{-2}\exp\left(-\frac{M+\Delta M}{M_{c}}\right).$$

Here, *M*_*c*_ is again the high-mass cut-off and Δ*M* is the average amount of mass lost from each GC. Clusters with (initial) masses less than Δ*M* have been fully disrupted. This simple formula describes the mass function of surviving GCs that started from an initial Schechter mass function (i.e. Δ*M*=0), under the assumption that the mass-loss rate is independent of cluster mass (this assumption is unlikely to be perfectly valid). For the Milky Way, a fit to the GC mass distribution yields Δ*M*=2.5×10^5^*M*_⊙_ and *M*_*c*_=8×10^5^*M*_⊙_.

If we assume that each of the *surviving* ≈100 halo GCs in the Milky Way has lost 250 000 *M*_⊙_, this amounts to about 2.5×10^7^*M*_⊙_. Within GCs, the enriched fraction is usually about 50%, so we may expect about 1.3×10^7^*M*_⊙_ of the stars that have been lost from surviving GCs to have enriched composition. It is interesting to compare this number with the amount of halo stars that display chemical abundance patterns typical of ‘enriched’ stars in GCs. Martell *et al.* [77] estimate that about 3% of the stars in their sample of 561 halo giants display enhanced N and depleted C abundances. If this fraction is representative of the halo as a whole, there should be about (1.2−2.1)×10^7^*M*_⊙_ of enriched stars in the halo. Considering the ‘back-of-envelope’ nature of this calculation, the similarity of these numbers is striking, and suggests that most of the chemically anomalous stars now observed in the halo field might have been lost from extant GCs. The above line of reasoning led Kruijssen [68] to conclude that the minimum cluster mass needed for survival over a Hubble time is similar to the minimum mass for hosting multiple populations. Whether this is a coincidence or emerges from similar physics is an open question. In reality, the enriched fraction in the stars lost from GCs may be lower than the present-day observed fraction within GCs, because the enriched stars tend to have a more centrally concentrated radial distribution. On the other hand, it would not be surprising if some fully disrupted clusters have contributed enriched stars to the halo.

The total current (*M*_cur_) and initial (*M*_init_) mass of a GC system can be found by integrating equation (5.1) over all masses, setting Δ*M*=0 for the initial mass. For a lower mass limit of 100 (5000) *M*_⊙_, the ratio *M*_init_/*M*_cur_ is 24 (13). Note that this does not include the factor approximately 2 that each GC will lose due to stellar evolution. For more realistic assumptions about the dynamical evolution, Kruijssen & Portegies Zwart [78] found factors of *M*_init_/*M*_cur_=64 (39), including the effects of stellar evolution. Several other authors have found *M*_init_/*M*_cur_>10 [76,79,80], implying that disrupted GCs might account for a significant fraction of the stars now belonging to the halo field. This provides an indirect constraint on the presence of multiple populations in the fully disrupted GCs: if they contained chemically anomalous stars in the same proportion as surviving GCs, these stars should now be present in the field in far greater numbers than are actually observed. Hence, it appears likely that if the present-day GC mass function is the result of dynamical evolution from an initial Schechter-like function, the fraction of chemically anomalous stars in the disrupted (primarily low-mass) GCs was lower than that in the present-day surviving GCs. This would be in accordance with the tendency for the enriched fraction to increase with GC mass [81], and the fact that multiple populations have not been found in open clusters.

Of course, it may be premature to assume that GC systems formed with a Schechter-like mass function. In particular, the large amount of mass loss required to turn a Schechter function into the present-day MF is in tension with the large fractions of MP stars that *presently* belong to GCs in dwarf galaxies like the Fornax dSph. At metallicities [Fe/H]<−2, 20–25% of the stars in the Fornax dwarf belong to GCs [82], and similar high fractions are found in the WLM and IKN dwarfs [83]. This appears difficult to reconcile with the idea that the present-day GCs should represent less than 10% of the initial mass of a GC population that initially followed a Schechter mass function. Alternatively, the initial mass distribution of GCs may have been more top-heavy, for example by shifting the lower mass cut-off to higher masses at the time of GC formation and/or invoking a shallower slope. Indeed, the young star cluster populations in some star-forming dwarf galaxies are dominated by one or two very massive clusters (e.g. NGC 1569, NGC 1705 and NGC 1023-A).

Of course, the high GC/field ratios in dwarf galaxies also pose a severe challenge for scenarios that invoke large amounts (90–95%) of mass loss from individual clusters in order to explain the large fraction of chemically anomalous stars within the GCs [84–87]. This challenge is greatly compounded if one additionally has to account for the dissolution of lower-mass GCs. It would be interesting to determine the fraction of enriched field stars in dwarf galaxies such as Fornax. If the GCs there have experienced significant mass loss, we might expect the enriched fraction to be much higher in the dwarfs than in the Milky Way halo.

### (b) The globular cluster mass function: nature or nurture?

The evolved globular cluster mass function (GCMF) mentioned in equation (5.1) does a good job in describing the luminosity function of Milky Way GCs, and those of GCs in external galaxies. But, we have yet to address the disruption mechanism that is responsible for shaping the GCMF. An important question is whether it is able to produce the correct turn-over mass (*M*_TO_), i.e. the value of Δ*M*, roughly independent of environment, as observed. Jordán *et al.* [76], Villegas *et al.* [88] and Harris *et al.* [89] discuss subtle dependencies of the shape of the GCMF on galaxy mass. Carretta *et al.* [64] show that the luminosity function of outer halo clusters contains slightly more faint (i.e. low mass) GCs than that of the inner halo clusters, and the luminosity function is similar to that of GCs in Local Group dwarf spheroidals. This higher frequency of low-mass GCs may point at evolution in weaker tidal environments.

Baumgardt & Makino [90] presented a suite of *N*-body simulations of GCs dissolving in a Galactic tidal field, which are still the default reference for GC evolution. The models consider GCs with a stellar mass spectrum, the effects of stellar evolution and orbits with various eccentricities in a Galactic potential that is approximated by a singular isothermal halo (i.e. *ρ*(*R*_G_)∝*R*^−2^_G_). They find that the mass-loss rate as the result of two-body relaxation-driven evaporation in a tidal field, ${\dot{M}}_{\mathrm{ev}}$, depends on *M* and the tidal field as $|{\dot{M}}_{\mathrm{ev}}|\propto M^{x}V_{\mathrm{circ}}/R_{G}$, where *V*_circ_ is the circular velocity in the galaxy and $\frac{1}{4}≲x≲\frac{1}{3}$. Using their results for the most massive GCs in their suite (approx. 10^5^ M_⊙_), and ignoring the small *M* dependence of ${\dot{M}}_{\mathrm{ev}}$ and adopting *V*_circ_≃220 km s^−1^, their results imply Δ*M*(*R*_G_)≃2.4×10^5^ M_⊙_ (kpc/*R*_G_) at an age of 12 Gyr. This means that at *R*_G_≃1 kpc the GCMF could have evolved from a −2 power-law function to a peaked function as the result of evaporation, but these models predict a decreasing *M*_TO_ with increasing *R*_G_. In the power-law regime of the GCMF, *M*_TO_∝Δ*M* [76], hence at *R*_G_≃10 kpc, *M*_TO_ would be a factor of 10 too low. Including the *M*^*x*^ dependence makes the discrepancy at large *R*_G_ even larger [91]. A concern with this argument is that beyond $R_{G}≳10\;\mathrm{kpc}$, the majority of the GCs are likely accreted from disrupted dwarf galaxies (see §3). We therefore need to understand the dynamical evolution of GCs within the framework of hierarchical galaxy formation to address the GCMF problem.

Typical values for the tidal field in dwarf galaxies are *V*_circ_≃10 km s^−1^ and *R*_G_≃1 kpc, such that *V*_circ_/*R*_G_≃10 Myr^−1^, i.e. comparable to the Milky Way tidal field at 20 kpc, i.e. a tidal field that is too weak to get the correct *M*_TO_ by evaporation. In addition, the (dark matter) density profile in dwarfs is flatter than −2, which further reduces ${\dot{M}}_{\mathrm{ev}}$ by a factor of 2 or 3 compared to a singular isothermal halo [92]. Finally, the adiabatic growth of the Milky Way brings GCs closer to the Galactic centre, such that ${\dot{M}}_{\mathrm{ev}}$ as derived from their present-day orbit overestimates the ${\dot{M}}_{\mathrm{ev}}$ averaged over their evolution in the Milky Way [93]. Therefore, the GCMF problem gets worse when invoking the hierarchical growth of galaxies.

Several models have tried to explain the universality of the GCMF by internal processes, such as the expulsion of residual gas [94] or stellar evolution [95], but these models make predictions for YMCs that have not been observed (see the review of [75]). McLaughlin & Fall [96] propose that evaporation results in mass loss on a relaxation timescale, such that $|{\dot{M}}_{\mathrm{ev}}|\propto \rho_{h}^{1/2}$, where *ρ*_*h*_ is the density within the half-mass radius. In this model, the details of the tidal field do not enter and this naturally results in an *R*_G_-independent *M*_TO_, and a correlation between *M*_TO_ and *ρ*_*h*_ is indeed observed. Although this model successfully explains the observations, the required mass-loss dependence on cluster properties—independent of environment—is inconsistent with results of numerical simulations of cluster evolution (e.g. [97,98]). Gieles *et al.* [99] show that a more plausible origin for the correlation between *ρ*_*h*_ and *M* is relaxation-driven expansion of low-mass GCs, rather than preferential disruption of high-density clusters.

Elmegreen [100] and Kruijssen [68] propose that interactions with giant molecular clouds (GMCs) in the first few 100 Myrs to Gyrs could efficiently disrupt low-mass GCs and shape the GCMF by dynamical evolution, but soon after GC formation. These ‘tidal shocks’ with GMCs disrupt the cluster on a timescale that is proportional to the density of the cluster [101]. This only results in mass-dependent disruption, if the density of clusters correlates with their mass [102]. This appears indeed to be the case for YMCs [103], which makes interactions with GMCs an additional disruption mechanism that can ‘turn over’ the GCMF. Elmegreen [100] and Kruijssen [68] adopt a weak mass-radius correlation (or a constant radius) and use estimates for the ISM properties at high redshift to argue that the strength of shock disruption is sufficient to get the correct *M*_TO_ within 1–3 Gyr. Gieles & Renaud [104] showed that the removal of mass by tidal shocks results in an increase of the cluster density, i.e. the disruption by tidal shocks is a self-limiting process. When considering the combined effects of tidal shocks (which shrinks clusters) and relaxation (which expands clusters), an equilibrium mass evolution $|\dot{M}|\propto M^{1/3}$ is found [104], similar to the mass evolution due to relaxation in a steady tidal field. We note that Kruijssen [68] already considered more compact clusters when studying the evolution of the mass function, in a way that is consistent with the decrease of cluster radii due to their response to tidal shocks from Gieles & Renaud [104], finding that tidal shocks are still able to turn over the GCMF. Whether the ISM at high redshift is sufficiently dense to evolve the GCMF with tidal shocks across the entire galaxy mass range, is an open question.

As some level of fine-tuning is required to obtain a near-universal GCMF with dynamical evolution it is tempting to consider an explanation that relies more on nature, rather than nurture. Indeed, several older models for the formation of GCs suggested that a typical mass of approximately 10^6^ M_⊙_ exists [60,61,105]. These were often based on cooling instability and Jeans mass arguments, and required fragmentation to produce lower mass GCs. More recent works (e.g. [106]), with updated cooling physics, suggest that the situation is more complicated than initially thought. We note that the majority of GCs in the Milky Way belong to the MP category, thus a formation model involving inefficient cooling at low metallicity is attractive. However, it is not clear if the observed floor in GC metallicity, i.e. no GCs have been observed with [Fe/H] <−2.5, can be reproduced by cooling instability physics. Molecular and atomic line cooling, which are dominant in the star-forming ISM, do not differ fundamentally from local-Universe cooling until much lower metallicities (e.g. [106]).

A way forward to address the long-standing problem of the GCMF is to expand the modelling efforts to include the radius/density of the clusters. The disruption by tidal shocks is efficient in destroying clusters of low density, whereas ${\dot{M}}_{\mathrm{ev}}$ depends only on *M* and the stationary tidal field. Fast cluster evolution models, such as EMACSS [107], guided by cosmological zoom-in simulations are ideally suited for enabling GC population synthesis studies that aim to reproduce the density of GCs in the mass, density, Galactocentric radius and [Fe/H] space.

## 6. How did globular clusters form?

Broadly speaking, the current literature contains two families of models for the formation and evolution of GC systems in the context of galaxy formation. The first family of models associates GC formation with special conditions in low-mass dark matter haloes, during or before reionization (e.g. [60,108–110]). The second family of models considers GC formation a natural byproduct of the active star formation process (which is often linked to high gas pressures) seen at high redshift [68,100,111]. In this latter branch of models, there is an ongoing debate between studies finding a relatively high importance of galaxy mergers in producing GCs [3,69,112] and those finding most GC formation proceeds in galaxy discs [68,70].

An end-to-end description of the assembly of GC systems during galaxy formation requires a model for star cluster formation in the early Universe, their initial mass function, their dynamical destruction (both in high-redshift environments by tidal perturbations from dense gas clouds and in low-redshift environments by evaporation), and their redistribution during hierarchical galaxy assembly and growth. The aforementioned two families of GC formation models differ in almost all of these ingredients and sometimes fundamentally so. Therefore, we briefly review each of these elements in the context of both model families, before discussing the different numerical approaches that are used by recent models, and discussing the key model predictions.

### (a) Globular cluster formation at very high redshift and connection to dark-matter halo assembly

GCs host some of the oldest stellar populations in our Galaxy and have been used in the past to constrain the age of the Universe (e.g. see [113]). Therefore, it is not surprising to see various investigations of formation scenarios at very high redshift, and connections between GC formation and dark-matter (DM) halo assembly. Early work by Peebles & Dicke [60] noted that the Jeans mass at high redshift (approx. 10^6^ M_⊙_) is comparable with the GC stellar mass, while other more recent studies focused on formation within DM mini-halos with characteristic mass *M*_DM_∼10^8^ M_⊙_. For example, Padoan *et al.* [114] suggested that compact star clusters can be formed through efficient H_2_ cooling; Cen [115] argued in favour of formation triggered by shocks induced by a hydrogen ionization front; Naoz & Narayan [116] investigated collapse of gas displaced by stream velocity from its parent DM halos; Trenti *et al.* [109] proposed instead that mergers of gas-rich but star-free mini-halos (*M*_DM_∼10^7^ M_⊙_) can lead to shock-induced compression and formation of central stellar clusters that would later be stripped of their DM envelope and become the population of old galactic GCs with a wide spread in metallicity but narrow difference in age. The appealing features of these models are that they are based on fundamental physical processes that are well established to happen during the early history of the Universe, at a time when the conditions for star formation were markedly different compared to those of low-redshift star formation because (i) the characteristic mass for collapsed structure was significantly lower, hence the massive disc galaxies that are frequently hosting young star clusters at lower redshift were extremely rare; (ii) the merger rate was significantly higher than that of the local Universe (e.g. [117]) as density scales with (1+*z*)^3^ and (iii) the ionizing background was lower (especially pre-reionization), but at the same time the higher cosmic microwave background floor was limiting the cooling of the gas to T≳30 K, potentially affecting characteristic mass and fragmentation of gas clouds (e.g. [118]). However, all proposed ideas are relatively qualitative, and further work is needed to develop them to the point where a data-model comparison is effective in falsifying them.

In addition, the typical mechanisms that are proposed for the formation of compact stellar systems are not likely to be in place at lower redshift, thus these classes of models explicitly (or implicitly) assume that younger GCs form through a different channel, even though recent observations show that young GCs may have properties that are indistinguishable from old GCs [65,66]. The existence of multiple channels could be physically motivated, because compact star clusters are forming in different contexts (e.g. nuclear star clusters, see [119]), but a single formation channel capable of explaining the broad range of GC system properties would arguably be preferred by Occam's razor. More broadly, current age measurements for Milky Way GCs are consistent with a significant fraction of GCs being formed during the first 800 Myr after the Big Bang (§2), but systematic uncertainties dominate the error budget, preventing a strong inference on observed ages. The key development that would allow for an efficient discrimination between models would be a technique to measure ages with uncertainties below 500 Myr to unequivocally establish what fraction of GCs (if any) are formed before the epoch of reionization at *z*>6.

A second feature broadly shared by models of GC formation in the context of DM halo assembly is that a characteristic mass scale for the formation process is identified. Therefore, the broad predictions/expectations are that the initial cluster mass function has a preferred scale, e.g in the form of a lognormal distribution, rather than a power law (e.g. [109]).

Finally, while some models argue for purely baryonic formation processes (e.g. [116]), the general feature of this class is that the dynamical assembly of the parent galaxy halo leads to tidal stripping of the DM envelope around GCs, which are expected to be characterized by high stellar densities (*ρ*_*c*_∼10^6^ M_⊙_pc^−3^) and compact radii (*r*_*h*_∼2 pc) if formed at *z*∼10 [120,121]. Under these conditions, it would be realistic to expect that even when the process is efficient, some GCs would still survive embedded in part of their DM sub-halo. Thus, high-resolution simulations that follow the complex gravitational dynamics of repeated mergers and stripping events would be needed to progress quantitatively to testable predictions.

### (b) Globular cluster formation as the natural outcome of star formation in normal high-redshift galaxies

In the local Universe, YMCs with masses and radii (and hence densities) very similar to GCs are observed to form in gas-rich environments of high star formation rate (SFR) and gas pressure (for recent reviews, see [21,75,122]). Given that the gas fraction, gas pressure, SFR density and SFR per unit gas mass in galaxies are lower at *z*=0 than in the early Universe, with the gas pressure and SFR peaking at *z*≈1−3 (e.g. [123–128]), it is therefore sensible to consider that GCs are the products of regular star and cluster formation in normal high-redshift galaxies. Indeed, this idea has motivated a broad range of models in which GCs form in gas-rich, high-redshift galaxy discs (e.g. [68,100,129]). In some variants, the formation of young GCs is enhanced by galaxy mergers (e.g. [69,112]). The simulations do not extend yet to *z*=0 and therefore cannot predict whether the clusters formed in mergers survive until the present-day (which at least for intermediate-mass clusters has been questioned in recent theoretical work, see [130]).

From a theory perspective, the conditions in gas-rich galaxies at high redshift are favourable to GC formation for two reasons. Firstly, the high turbulent pressures are predicted to lead to a high fraction of star formation occurring in gravitationally bound clusters ([131]; also see [132]), which follows the same trends as observed in actively star-forming galaxies in the local Universe [133,134]. Secondly, the high gas pressures in the turbulent interstellar medium (ISM) of these galaxies also imply high maximum mass scales for gravitational collapse (e.g. [135,136]), which potentially enables the formation of massive GMCs if they overcome shear, centrifugal forces and feedback (e.g. [137]). Some fraction of these GMCs may form massive clusters (e.g. [138]). In the optimal case, the resulting maximum cluster mass scales linearly with the GMC mass, modulo factors corresponding to the star formation efficiency and the fraction of star formation that ends up gravitationally bound [21]. Observations and theory show that the resulting maximum cluster mass increases with the gas pressure (which may be traced by the gas or SFR surface density, [139,137]). In the highly turbulent and high-pressure environment at high redshift, this plausibly leads to the formation of very massive stellar clusters that can remain gravitationally bound over a Hubble time.

It is not yet clear what the role of galaxy mergers is in determining the properties of the GC population at *z*=0. Galaxy merger rates are orders of magnitude higher at high redshift (*z*>1) than at the present day (e.g. [117]) and could facilitate the assembly of very large GMCs (either by direct cloud collisions or by raising the ISM pressure), which could host more massive star clusters than in discs (e.g. [69,112]). While the conditions in galaxy discs may already be sufficient to form GCs, mergers could therefore enhance GC formation. However, mergers might also increase the destruction rate of star clusters [130] due to the increased gas densities (see below). In addition, the fraction of all star formation occurring in mergers may be low, of the order 10% [140]. In the view of these uncertainties, a detailed quantification of the contributions of mergers to GC formation is needed. Even if mergers do not represent a large fraction of all GC formation, they may eject GCs into galaxy halos where they can survive over longer timescales [129,68].

It is now widely accepted that regular star cluster formation in galaxy discs leads to a power-law initial cluster mass function with a gradual (e.g. exponential) truncation at the high-mass end; pure power laws are ruled out [69,75,139,141,142]). Also, the GC mass function has a similar truncation at the high-mass end [68,76,79]. Therefore, the favoured model for the initial cluster mass function during regular star formation in high-redshift galaxies is a Schechter [143]-type function. We note that lognormal initial cluster mass functions are inconsistent with the hierarchical ISM structure observed in galaxy discs (e.g. [144,145]). In the specific context of GC formation as the natural outcome of high-redshift star formation that is considered here, the ICMF should thus follow a (truncated) power law. In a broader context, this is a general result of the scale-free structure of the ISM. However, the exact form of the GC initial cluster mass function remains unknown until it has been observed directly.

Even if GC-like objects form at high redshift, they need to survive till *z*=0 in order to be observed as GCs. Models and simulations accounting for this process need to include a variety of mass loss and destruction mechanisms, such as stellar evolution, tidally limited evaporation, time-variable tidal perturbations (tidal shocks) due to interactions with, e.g. the ISM or galactic structure, and dynamical friction. Stellar evolutionary mass loss equally affects star clusters of all masses, but evaporative and tidal shock-driven mass loss mostly affects low-mass clusters. Theoretical and numerical studies find that tidal shocks represent the dominant destruction mechanism in the gas-rich environments where star clusters form [102,146–149]). As a result, it is a key requirement for star cluster survival to escape its gas-rich formation environment, possibly by galaxy mergers or other (gas or cluster) migration mechanisms [129,68]. To date, few models for the origin of GCs in the context of galaxy formation have included tidal shocks, barring a couple of exceptions [68,70,100]. It is important to note that the efficiency of tidal shocks depends on the spatial resolution of the simulations. It always represents a lower limit unless the high-mass end of the GMC mass function is well resolved, because a GMC mass function with a slope shallower than −2 implies that most of the destructive power should come from high-mass ISM structures.

In contrast to the other mass loss mechanisms, dynamical friction destroys the most massive star clusters. This has an important implication in the context of direct detections of young GCs with next-generation telescopes such as JWST (see §2). While the most massive GCs are likely to survive till *z*=0 against disruption by evaporation and tidal shocks, they are the least likely to survive inspiral by dynamical friction, provided that they formed in the inner parts of their host galaxy where most of the dense ISM resides. A 10^7^ M_⊙_ cluster (which is just three times more massive than the present-day GC truncation mass) in a galaxy with a circular velocity of 100 km s^−1^ has a dynamical friction timescale of *t*_df_∼5 Gyr for an initial galactocentric radius of *R*∼5 kpc, with *t*_df_∝*R*^2^, highlighting that sufficient time is plausibly available for this mechanism to affect the most massive GCs and to have set the maximum mass scale observed today. It is therefore unclear if the massive clusters observed with JWST will survive to become GCs at *z*=0; the identification of star clusters at high redshift as proto-GCs is challenging, because it requires accounting for the above disruption mechanisms.

When considering the balance between formation and destruction mechanisms, there have been ongoing debates whether the observables describing the GC population at *z*=0 are shaped mostly by formation or by disruption. Examples are the peaked GC mass function, which is an imprint of formation in GC formation models related to dark matter halos and reionization, whereas it is the result of GC disruption in the ‘regular cluster formation’ family of models. Likewise, the specific frequency (number of GCs per unit galaxy mass or luminosity) has been referred to as a GC formation efficiency (e.g. [150,151]), whereas recent work argued that it mostly represents a survival fraction [68]. The same question has been discussed in the context of the constant GC system mass per unit dark matter halo mass (e.g. [152–154]), which has been suggested to be the result of self-regulation by feedback from the host galaxy [155,156], GC formation proportional to halo mass (e.g. [108]), or as a coincidental combination of the opposite galaxy mass dependences of the GC survival fraction and the baryon conversion efficiency, with possibly an additional linearizing effect of the central limit theorem during hierarchical galaxy assembly [68]. Finally, it is relatively broadly supported that the GC metallicity distribution largely traces formation, in the sense that MP GCs mostly formed *ex situ* in low-mass galaxies, whereas MR GCs mostly formed *in situ* within the main progenitor [157].

## 7. Numerical approaches to model globular cluster formation during galaxy formation

Attempts to describe GC formation mechanisms as an integral aspect of galaxy formation are rapidly increasing. Here, we briefly summarize the different modelling approaches in broad categories and provide a short description of their benefits and shortcomings.

### (a) Extremely high-resolution cosmological zoom-in simulations

As the ongoing development of computing algorithms enables galaxy formation, simulations reach parsec-scale spatial resolution, it opens a new avenue to study GC formation by resolving the process directly. This requires particle or cell masses below 10^3^ M_⊙_ and is computationally extremely demanding. As a result, it is presently very difficult to follow the system to *z*=0 and current examples in the literature therefore stop the simulation at a higher redshift. The major advantage of this approach is that the formation of clusters is based on a higher degree of ‘first principles’ physics. Still, some important physical mechanisms remain uncaptured, most critically so in the area of star cluster mass loss, because the collisional stellar dynamics that drive evaporation and determine the cluster density profile (and hence its response to tidal shocks) are unresolved. It is also not possible to know if the star cluster may fragment into smaller systems when increasing the resolution. However, direct high-resolution simulations are the best avenue for testing and exploring the physics of massive star clusters in high-redshift environments.

Kim *et al.* [112] present the first cosmological hydrodynamic simulation of the formation of a massive star cluster (*M*∼10^6^ M_⊙_) at redshift *z*≈7, resolved with many (approx. 10^3^) stellar particles. Similarly, Li *et al.* [69] model the formation of GMCs in cosmological simulations with about 5 pc spatial resolution, and the growth of cluster particles through gas accretion that is terminated by self-consistent feedback. This method enables the particle masses to be interpreted as cluster masses and tests the simulation results against the observed cluster mass function. The simulation finds good agreement, with a power-law initial cluster mass function and an exponential truncation at the high-mass end.

### (b) Subgrid modelling in self-consistent galaxy formation simulations

The second method for modelling GC formation and evolution during galaxy formation is to use subgrid models. The major advantage of this approach is its modest computational cost and, as a result, the greatly improved statistics of the modelled GC population, while retaining some description of the pertinent physics. These models typically adopt numerical resolutions similar to other galaxy formation simulations and, as a result, can comfortably run to *z*=0, enabling comparisons between the modelled and observed GC populations. In addition, the shorter run time enables carrying out large numbers of simulations that systematically explore the effects of the subgrid models describing cluster formation and evolution, providing insight into which physics matters most in shaping the GC population. This method also opens up the possibility of simulating a range of galaxies with a variety of formation and assembly histories, thus probing the impact of these histories on the properties of the GC population and thereby fulfilling the potential of GCs as tracers of galaxy formation and evolution. The relatively low computational cost also permits simulating volumes larger than Milky Way-mass galaxies, feasibly up to Virgo cluster-like masses with current computational facilities. The obvious downside of this approach is that subgrid models require compromises in terms of the accuracy and level of detail at which the formation and disruption physics are described. However, the fact that at least some physically motivated description of the GC formation and disruption physics is included implies that this approach is currently the only method that provides an end-to-end description of the formation and evolution of the entire GC population during galaxy formation, from high redshift till the present day, while capturing the environmental dependences and complete population statistics.

Initially, the modelling efforts in this direction post-processed dark matter-only simulations or the results thereof by adding baryons through observed scaling relations and analytic physical prescriptions and inserting GCs at particular epochs. These models are largely (semi-)analytical in nature (e.g. [1,2,68,158]). More recently, self-consistent hydrodynamic simulations of galaxy formation are used to model the formation and destruction of the GC population over long (from many Gyr to a Hubble time) timescales [4,70,130,148]. Both branches of approaches highlight the major impact of environment on the GC system properties, implying that accounting for the galaxy formation and assembly history is crucial for understanding GC formation and evolution. An important consequence of this variance in GC systems is that a single simulation of a single galaxy is unlikely to provide widely applicable insights—statistical samples of galaxies with a range of assembly histories are needed to come to more generally applicable conclusions.

### (c) Particle tagging and dark matter-only simulations

The third approach by which the formation and assembly of GC systems is studied is by particle tagging in (possibly dark matter-only) galaxy formation simulations. Tagging particles and representing these as GCs is the most phenomenological approach out of the three methods described here, which is why it has been popular for more than a decade (see e.g. [159], but also more recent examples by Corbett Moran *et al.* [160], Mistani *et al.* [161] and Renaud *et al.* [3]). Its most obvious advantage is the major versatility in selecting which subpopulation of particles to tag, and the fact that the tagging can be performed in post-processing, whereas the two other methods discussed above require on-the-fly modelling. The downside is that models relying on particle tagging do not include a self-consistent treatment of the relevant physics and will yield GC systems that are biased towards the tagged (e.g. field star or dark matter) population. They also have trouble to self-consistently capture cluster disruption for all clusters across a large sample of galaxies due to the prohibitively large amount of storage space required.

The above limitations are particularly relevant because the environmental dependence of GC formation and disruption physics implies that the spatial distribution of GCs at each redshift matters for their properties at *z*=0. For instance, the specific frequency of GCs varies by up to three orders of magnitude across the GC metallicity range [162], suggesting that, combined with the stellar metallicity gradient in galaxies, on average, field stars do not occupy the same distribution in six-dimensional (position-velocity) phase space as GCs. Therefore, the results of particle tagging methods will be sensitive to the exact subpopulation of particles that is tagged. Likewise, if satellite dark matter haloes are tagged by following the observed spatial profiles of GCs (as in Moore *et al.* [159]), it suggests that GC formation redshifts that are too high to be consistent with the observed GC ages (cf. [163,6]). Most likely, this discrepancy arises because multiple GCs could form per satellite halo and because the spatial profiles of GCs were affected by environmentally dependent GC formation efficiencies and survival rates.

Despite the various caveats that should be kept in mind when using particle tagging to model GC systems, this approach can lead to valuable insights when physically motivated, specific subsets of particles are tagged. For instance, Tonini [157] used dark matter-only simulations to demonstrate how bimodal metallicity distributions of GCs can arise due to the accretion of dwarf galaxy satellites. Renaud *et al.* [3] showed that the old star particles (greater 10 Gyr) in a cosmological zoom-in simulation have a similar density distribution and kinematics at low redshift as found for the Milky Way's oldest GCs. This suggests that GCs may follow the earliest phases of star formation, where some (yet to be understood) fractions of the stars end up in GCs. Such studies should be followed up with detailed subgrid models incorporating GC formation and evolutionary processes.

## 8. Prospects for modelling the formation and co-evolution of globular clusters and their host galaxies in a cosmological context

In order for cosmological hydrodynamical simulations of galaxy formation to prove an informative tool with which to study GC formation and evolution, an obvious prerequisite is that they should reproduce key observed scaling relations of the galaxy population. Only in recent years have such calculations begun to satisfy this condition, largely due to significant developments in the modelling of ‘feedback’ processes (e.g. [164–166]) that regulate (and even quench) galaxy growth by (re)heating and ejecting cold gas from galaxies.

Owing to the dynamic range of the problem, the scope of cosmological simulations is often limited by their memory footprint, and those that follow volumes sufficiently large to realize a representative population of galaxies typically achieve a spatial resolution of only around 1 kpc. This precludes detailed numerical modelling of the life cycle of the ISM, motivating instead phenomenological treatments of star formation (e.g. [167–169]) and feedback (see e.g. [170–178]).

The use of such treatments has shown, however, that the inclusion of feedback does not necessarily lead to the formation of realistic galaxies; simulations incorporating well-motivated feedback prescriptions can yield galaxy populations with unrealistic masses, sizes and kinematics (e.g. [179,180]). The systematic study of Scannapieco *et al.* [181] found that leading hydrodynamical models, when applied to identical initial conditions of a halo much like that of the Milky Way, yielded present-day stellar masses that varied by almost an order of magnitude. It is likely the case that much of this dispersion stems from unintentional numerical losses of the injected feedback energy (for example, in the regime where the local cooling time of a gas element is shorter than its sound crossing time, e.g. [174]), and differences in the assumed physical efficiency of the feedback. The dispersion serves to illustrate the sensitivity of the global star formation history of galaxies to the efficiency of feedback. Recognition of this sensitivity has motivated exploration of the impact of varying the properties of feedback-driven outflows in response to the growth of the galaxy, which has been found to be an effective means of governing star formation histories (e.g. [182–184]). This has led to the development of simulations that explicitly calibrate feedback efficiencies to reproduce key low-redshift observables such as the galaxy stellar mass function from Illustris [185] and from EAGLE simulations [186,187]. Encouragingly, these simulations also reproduce many other diagnostic quantities that were not calibrated.

Realistic galaxy formation simulations present a promising foundation from which to explore the formation and co-evolution of GCs and their host galaxies. Renaud *et al.* [3] examined a high-resolution cosmological simulation of a galaxy evolved to *z*=0.5. Tagging stellar particles as potential star clusters, they computed the tidal forces these particles were subjected to throughout the assembly of the host galaxy. They showed that the tidal forces experienced by stellar particles evolve markedly over cosmic timescales, and as a function of a particle's (evolving) location within the galaxy and its progenitors. The E-MOSAICS project [4,70] couples the analytic MOSAICS models [148,130] of star cluster formation and evolution to the EAGLE galaxy formation model, initially presenting results from 10 cosmological zoom simulations of typical present-day disc galaxies. Modelling the number density and initial properties of star clusters associated with each stellar particle, these simulations indicate that the physics governing star cluster formation results in cluster formation histories deviating markedly from the formation histories of field stars [188]. Like Renaud *et al.* [3], the E-MOSAICS simulations compute the tidal forces acting upon star clusters throughout the simulation, but in addition use these measurements to estimate the rate at which star clusters are tidally disrupted, in a fashion similar to the scheme of Prieto & Gnedin [189].

Cosmological zoom simulations likely represent the optimal compromise between the competing requirements of detail and diversity. Although they typically focus on the evolution only of an individual galaxy, they model the cosmic environment experienced by all of the star clusters that form within its progenitors. Having a relatively small memory footprint, multiple zooms can often be run with relatively modest computational resources, enabling diverse samples of galaxies to be assembled. Recent zoom simulations of typical disc galaxies evolved to *z*=0 have realized force resolutions of around 10–100 pc [190–193], while zoom simulations of dwarf galaxies have achieved around pc force resolution (e.g. [194,110]). Such resolution is clearly still some way from that necessary to numerically integrate the internal dynamics of star clusters, but it is adequate to move beyond the widely used phenomenological treatments of star formation that are calibrated to reproduce the observed Schmidt (or Schmidt–Kennicutt) relation (e.g. [195]), which relates the gas density (or projected gas density) and star formation rate (or projected star formation rate) integrated over galaxies or star-forming regions. For example, Semenov *et al.* [196], building on work by Schmidt *et al.* [197], compute the local star formation efficiency per free fall on around 10 pc scales using subgrid turbulence models, the latter being calibrated against high-resolution simulations of star formation in the super-sonic MHD simulations of Padoan *et al.* [198]. This approach allows for a non-universal star formation efficiency in GMCs, as is observed (e.g. [199]), and may be important for realizing the very high efficiency of star formation in the densest peaks of the ISM.

Similarly, at such high resolution, it becomes necessary to move beyond stellar feedback treatments that instantaneously inject energy and/or momentum from an entire simple stellar population. Stellar evolution codes can be used to predict the injection rate of energy and momentum from radiation, stellar winds and SNe over the lifetime of a population [176,192]. Moreover, high-resolution simulations are beginning to offer meaningful predictions of the coupling efficiency of SNe in turbulent media (e.g. [200–202]) which, in principle, can be parametrized and adopted as subgrid models in cosmological simulations [190–192,203].

The dynamic range required to model star formation explicitly in a broad cosmological context will remain beyond the scope of state-of-the-art computational resources for many years to come, and hybrid approaches marrying numerical and (semi-)analytic techniques will likely remain the most profitable line of enquiry. By pushing the interface between the two components to ever smaller scales, the influence of restrictive approximations within the latter can be minimized and, in principle, the predictive power of the models increased. Pursuit of this route, however, requires that two key challenges be addressed. Firstly, numerical astrophysicists must remain able to capitalize on the increasing capacity of high performance computing facilities, which are increasingly delivering increased computing power via greater parallelism (i.e. more processors, and more cores per processor) rather than greater processor clock speeds. Secondly, the more detailed physics governing the life cycle of interstellar gas, such as molecular chemistry (Kim *et al.* [112]) and the formation of dust grains, radiation transport from local sources within the ISM, magneto-hydrodynamical effects and the transport of cosmic rays, must be incorporated into hydrodynamic simulations in a realistic fashion. The lack of convergence in the implementation of relatively simple phenomenological feedback schemes, highlighted by the study of Scannapieco *et al.* [181], serves as a reminder that this challenging step is likely to remain frontier science for some time to come.

## 9. Conclusion

GCs continue to be a topic of active research with many fundamental questions remaining. These include: How and when did GCs form? Where did they form and how did they assemble into today's GC systems? What was their initial mass function? Answers to these questions will come from both observations and simulations.

Most GCs of the Milky Way, and those around external galaxies, are very old. Although indications are that the MR GCs are approximately 1–1.5 Gyr younger than the MP ones, it is still difficult to rule out conclusively coeval ages for the two subpopulations. Absolute ages place the oldest GCs at around 12.5 Gyr but uncertainties extend their ages well into the epoch of reionization (*z*>6).

Near-term prospects for making progress with absolute ages for resolved GCs include obtaining near-IR colour magnitude diagrams that exploit the age sensitivity of the main sequence turnoff and a feature associated with H_2_ opacity. For unresolved, but nearby, GCs measuring relative ages with the next generation of wide-field, multiplexing spectrographs will be very challenging with 8–10 m telescopes. Progress will probably have to wait for large numbers of GCs to be observed with the 20–40 m class telescopes and will need to be accompanied by a better understanding of theoretical integrated-light age diagnostics. An exciting new avenue, which has recently been opened up by HST, and will no doubt be followed up by JWST, is the direct observation of proto-GC candidates at high redshift. Some fraction of these objects may have survived destruction processes to comprise the present-day population of GCs.

Multiple populations (within an individual GC) appear to be a common feature in old GCs—indeed, they may be a defining feature of a GC. Recently, all the hallmarks of multiple populations have been found in compact, massive star clusters of age around 2 Gyr. Thus, the conditions necessary for multiple populations exist in the local universe. These young GCs, forming at *z*∼0.17, suggest a common single channel for GC formation.

Owing to internally and externally driven destruction processes, GC systems formed with much more mass than is observed today. The commonly discussed formation models for multiple populations require that all individual GCs were at least ten times more massive than they are currently in order to solve the mass budget problem (i.e. to have enough material processed through a first generation of stars to form a second generation of stars that matches the present enriched fraction ratio). However, this universal amount of mass loss is not expected from models of GC evolution; instead, it comes from fine-tuning the initial conditions in order to match the observed population ratios. Relatively extreme initial conditions must be assumed, and the present-day GC population properties are not consistent with these assumptions. For the Milky Way, assuming a power-law mass function, the initial mass of the entire GC system has been estimated to be 10–60× that of its current mass (few around 10^7^ M_⊙_). For dwarf galaxies, such large inferred mass losses are in tension with the large fraction of MP stars in GCs. The initial shape of the mass function for old GCs is currently unknown—it is still an open question as to whether the initial mass function resembled a power law or had a preferred mass scale. However, for massive galaxies a power-law initial mass function is currently favoured. These issues remain areas of active research.

The marked influence of environment on the evolution of GCs requires that self-consistent models consider GCs within the full cosmological context of their host galaxy. Clearly, such models are also a necessity when seeking to explore and interpret the observed correlations between GCs and their hosts. The dynamic range posed by this necessity in terms of the mass, length and timescales—particularly when modelling populations of GCs—presents a major challenge to the modelling community. Moreover, the goalposts are likely to move again in the near future, as the promise of JWST is realized. However, a number of ambitious, complementary modelling approaches have recently emerged, which have demonstrated plausible origins for key present-day GC-galaxy scaling relations. These recent successes should foster genuine optimism for the discovery potential of the forthcoming generation of cosmological simulations of GC systems.

## Data accessibility

This article has made use of NASA's Astrophysics Data System (ADS).
